# A study of Chinese medical students’ communication pattern in delivering bad news: an ethnographic discourse analysis approach

**DOI:** 10.1186/s12909-021-02724-6

**Published:** 2021-05-19

**Authors:** Jack Pun

**Affiliations:** Department of English, City University of Hong Kong, 83, Tat Chee Avenue, Kowloon, Hong Kong SAR, China

**Keywords:** Medical Students, Communication, Bad News, Scenario, Ethnographic Discourse Approach

## Abstract

**Background:**

Breaking bad news is inevitable for prospective doctors, it is important for medical students to learn how to humanely communicate devastating news to patients. This study explores the discourse strategies used by Chinese medical students when conducting critical conversations via role-play scenarios.

**Methods:**

Fifty Year-6 medical students attending the ‘Serious Illness Communication Module’ were recruited from a local medical school in Hong Kong. They were asked to participate voluntarily in two role-play scenarios requiring them to break bad news to a simulated patient in Cantonese. The verbal interactions were video-recorded and analysed using an ethnographic discourse approach to unpack the quality of the observed interaction sequences and identify the discourse strategies strategically used by the medical students to overcome any communication breakdowns (e.g. linguistic expressions conveying diagnoses) and show empathy to patients.

**Results:**

Six discourse strategies for delivering bad news were identified in the Chinese context: (1) placing great emphasis on patients’ emotional needs; (2) informing patients with a balanced focus on medical and emotional needs; (3) directing patients’ attention to treatment options; (4) acknowledging concerns about dying patients’ physical discomfort and wishes; (5) directing bad news disclosure to patients; and (6) addressing the family expectations of patients. The majority of the Chinese medical students in this study used a patient-oriented approach to cater to the patients’ emotional and physical needs. They also often informed and acknowledged the patients’ family members.

**Conclusions:**

When delivering bad news, medical students should be equipped with discourse strategies that effectively balance interpersonal communication with the communication of medical expertise, which is integral to ensuring patients’ participation, their understanding and satisfaction with their clinicians. This is in accordance with the existing communication frameworks for critical conversation and demonstrates awareness of the needs in the Chinese context. However, some students demonstrated poor sensitivity to non-verbal cues, such as tone, manners and attitude. Thus, more training using a culturally appropriate model of   communication for critical conversation should be promoted.

## Background

Delivering bad news is indispensable to doctors’ daily work. Doctors are required to engage in conversations about sensitive topics such as disability, end-of-life care, prognostic uncertainty, or even death. Yet the bad news delivery context can be a challenging and complex communication task. Apart from the verbal component of actually passing on the message, it requires a range of other communication skills. These include making appropriate responses to patients’ emotional reactions, engaging patients in the decision-making process, managing stress arising from patients’ expectations, communicating with family members, and dealing with the difficulty of giving hope in a bleak situation [1]. Due to this complexity, doctors frequently report emotional intensity, communication difficulty and being underprepared for the responsibility when breaking bad news [2–4]. Their fear towards bad news delivery include being criticized, the unknown and untaught, managing patients’ reactions, not knowing all the answers, illness and death, which may result in emotional disengagement from patients [5, 6]. Moreover, insensitively or inadequately conducted discussion could pose serious challenges to patients’ and their family’s long-term adjustments to the outcome of the bad news [7].

Considering the importance and complexity of bad news delivery, specific communication training for both doctors and medical students is in great need. In response to this need, several protocols and frameworks have been developed and tested in existing literature. For example, Villagran et al.’s COMFORT model (an acronym for communication, orientation, mindfulness, family, ongoing, reiterative messages and team), which is a set of guiding principles providing a step-by-step guide on how to break bad news while providing comfort and remaining humane [8]. It proposes a set of skills that should be rendered concurrently by patients, family members and clinicians. SPIKES (an acronym for setting, perception, invitation, knowledge, empathetic response and summary) is a six-step protocol that helps doctors properly prepare to deliver bad news to their patients and ensure their patients’ comfort and understanding of the issue [9].

While communication protocols and frameworks are increasingly integrated into Western medical education, such approaches as those outlined above may not be applicable to bad news delivery communication in non-Western cultures [10]. This is because sociocultural factors play an important role in patients’ communication preferences and understanding of emotionally challenging issues such as end-of-life care and death [11, 12].

In the Chinese context, traditional beliefs and taboos have an important bearing on bad news delivery discussion. One of the key sociocultural factors is the traditional philosophy of Confucianism, which highlights familial responsibility and harmony [13]. This tradition, combined with collectivism and filial piety valued by Chinese society make  decision-making a family-centred practice as family is heavily involved in decision-making and the development of treatment plan. Consequently, healthcare providers are expected to talk to family members first, who then deliberate on whether to tell the patient [14]. Another challenge clinicians face is the traditional consideration of death as a taboo topic in Chinese culture. Instead of talking about death openly, the Chinese tend to use euphemistic expressions or avoid the topic altogether, making delivering bad news harder for doctors and medical students working in the Chinese context [15]. Thus, doctors should be equipped with both effective communication skills and increased awareness and sensitivity to patients’ needs in specific sociocultural contexts.

Some communication frameworks have been modified and adapted for the Chinese cultural context, such as the Calgary-Cambridge Guide and the SEGUE (set the stage, elicit information, give information, understand the patient’s perspective and end the encounter) framework [12, 16, 17]. The SEGUE framework, which stemmed from Western ideologies have yet adjusted to tailor to particular Chinese cultural elements such as collectivism, aims to facilitate practitioners’ communication skills and reflect cultural differences between Chinese society and the West [18]. However, they do not provide any communication patterns that medical students, especially those with limited clinical experience, to address patients’ emotional needs as underpinned by their cultural backgrounds. In addition, communication training programs in the Chinese context are limited in both medical schools and hospital settings, which mainly emphasise training medical students to communicate medical information rather than to address patients’ emotional needs (for an overview, see Liu et al., 2015) [19]. None of the standardised communication protocols consider local culture in understanding and interpreting medical encounters, which may significantly affect the relationships between doctors and patients.

To address the literature gap, this study explores Chinese medical students’ communication patterns when conducting critical conversations in role-play scenarios. The findings can guide the future of medical programmes and trainings to provide medical students with a bad news delivery communication framework that addresses the specifics of Chinese cultural contexts and improves patient relationships.

### Methods

#### Study design

Medical students’ communication patterns during bad news delivery were examined in two scenario-based role-play activities. Simulated role-play activities provide medical students an opportunity to understand patients’ views in critical conversations, to experience the communication challenges doctors may face in breaking bad news and to explore the appropriate language responses in local language.Video-recorded role-play allows students to reflect on their verbal and non-verbal communication practices in handling critical conversations. It can also facilitate peer-review and learning. Video-informed role-play is also an effective teaching method for communication skills training. Thus, the simulated role-play activity was recorded to allow careful analysis of and reflection on the students’ verbal and non-verbal communication practices.

#### Data design and collection

Fifty Year-6 students from a local medical school were recruited to participate in the role-play sessions. All of the student participants attended four sessions of the ‘Serious Illness Communication Module’ to ensure that they had a certain level of knowledge about communicating with patients diagnosed with serious illnesses. All students participated voluntarily based on their willingness and could opt out when they feel necessary. Two actors were hired to portray patient’s relative or a senior consultant. Two role-play cases of medical students’ breaking bad news were video-recorded along with field notes, with each role-play session lasting for 10 mins. Each group of participants consisted of a patient relative/carer (portrayed by an actor), a doctor, a senior consultant (portrayed by an actor) and one or two observers (including the researcher) to explore one of  two challenging situations faced by the patients or patients’ relatives. Finally, a focus group discussion was organized by the course instructor, who is a geriatrician with experience in teaching clinical communication in Hong Kong. The participants’ reflections on their communication practices were audio-recorded and analysed according to their emerging themes. The communication patterns for handling their patients’ emotions and effectively engaging with them in difficult conversations were identified.

### Data analysis

All of the spoken interactions during the bad-news-delivery role-play scenarios were video-recorded and detailed observation notes were made. All collected data were transcribed and translated into English. The videos and transcripts were verified by two clinicians, one critical care physician and one geriatrician, who both are practitioners and clinical educators. In addition, a research assistant from the team double-checked all the transcriptions and translation against the video recordings and observation notes. A coding framework was developed after two rounds of discussions among the researcher and the healthcare professionals. About 10 % of the data was independently coded by two raters to ensure reliability of the coding framework. The current qualitative cross-sectional observational study follows the Strengthening the Reporting of Observational Studies in Epidemiology (STROBE) protocol as described by Von Elm [20].

The researcher has been working closely with doctors to investigate the quality of health communication in different medical contexts as well as developing and implementing communication training programs for healthcare professionals and medical students. In collaboration with the course teachers, a number of communication patterns were identified. Specific attention was paid to the types of information presented and how the medical students delivered bad news.

An ethnographic discourse analytical approach was used. The combination of discourse analysis with ethnographic methodology takes reference from Slade et al. [21] where researchers can enrich their interpretations of observed clinical practices, such as by explaining how language is used between a doctor and patient to construct meaningful communication and achieve specific institutional and clinical goals. The quality of the interaction sequences between the medical students and the simulated patients was unpacked. Their discourse features including but not limited to how the students initiated, responded to and followed up with the patients’ answers were identified. Students’ language use such as question types, turn-taking and pauses in the observed interaction was identified and their communication patterns were categorised. Particular attention was paid to how the students explained clinical diagnoses and treatments and the expressions they used when describing symptoms during bad news delivery. Furthermore, how students responded to patients’ emotional needs (e.g., how they answered the patients’ questions) and patients’ requests for clarification was explored. The ways medical students elicited the patients’ concerns about both their medical and psychological needs could be identified using this method.

The following section presents the findings of the video-recorded role-play scenarios. It highlights the communication patterns in delivering bad news, that is, how diagnoses were communicated and how medical students and patient actors collaborated for effective communication and decision-making.

## Results

### Communication patterns for delivering bad news in the Chinese context

The role-play sessions allowed for a thorough examination and analysis of the medical students’ communication patterns in communicating with patients. Six patterns were identified: (1) greatly emphasising patients’ emotional needs; (2) showing insufficient care about patients’ emotional needs; (3) prioritising the preferred treatment plan instead of respecting the needs of patients’ families; (4) acknowledging dying patients’ physical discomfort and respecting their treatment preferences; (5) directly disclosing bad news to patients; and (6) addressing patients’ familial requirements.

#### Greatly emphasising patients’ emotional needs

The first communication pattern of greatly emphasising patients’ emotional needs is demonstrated in Table 1. The medical student shows high awareness of the patient’s emotional needs and makes them a priority, choosing to deal with those needs before providing factual information about treatment plans. In the transcript below, the patient expresses her concern about not consulting a doctor sooner and self-blames as a result (line 38). The medical student recognises the patient’s emotional need for reassurance (line 39) and acknowledges the patient’s unhappy and guilty feelings. The medical student also redirects the patient’s train of thought, persuading her to discuss possible treatment plans instead of thinking about what she could have done in the past, preventing possible further aggravation of the patient’s mental state. This is because if the patient continues to think about their past decisions in the treatment process, they might experience further distress and guilt. This awareness of the patient’s emotional need for reassurance is also observed in the medical student’s declaration that he/she will do his/her best to help the patient and that the patient can find him/her anytime to talk about her condition (Table 1).

**Table 1:** First transcript about emphasising patients’ emotional needs

38	Patient	So perhaps I should have consulted a doctor earlier? I had already been coughing and had felt unwell for quite a while. Does it mean that if I had consulted a doctor earlier…
39	Medical Student	Um, so I can tell that you might be feeling quite unhappy right now and also are blaming yourself a little bit, but we shouldn’t think about things the way we are thinking now. How about we come up with something that can make you feel better? Then you will feel less suffocated and you will feel less pain in your chest. So, we will, um, heal you and we want to help with whatever we can. So, let’s see and think about what can help, help with your suffering, just like that, yeah. And also heal you up. Then, as for how, and, um, erm, as for what you said about seeing the doctors earlier could have prevented this from happening, actually, actually, at this moment we, it’s better for us not to think about it, then we can, then we can at this moment, I mean how about we focus on how to cure your disease now, is that okay?Ah yes, you can find me any time, erm, if you have any questions that you want to ask me, you can get these nurses to let me know, I will, erm, also do my best of course. That means if I am free, then I will, erm, as soon as possible, erm, reply and talk to you about that, your condition, I mean.

In  Table  2 below, the medical student demonstrates care for the patient’s emotions by providing a reassuring answer to the latter’s question regarding his/her chances for a full recovery (line 30). The medical student not only focuses on the actual probability of the patient being fully cured, but also on comforting the patient. In line 31, the medical student breaks the bad news that the patient may not be fully cured and immediately reassures the patient and shows care for his/her emotions. The student tells the patient that the entire hospital will endeavour to assist him/her and that the medical team will not abandon him/her. These two statements help prevent the patient from becoming distressed due to the bad news and give him/her a sense of support. The student tells the patient that he/she can contact the staff anytime if her/she has any questions or needs. This statement helps reinforce the patient’s sense of support and makes it clear that the patient’s needs are prioritised by the staff. In the above communication style, the patient is repeatedly reassured and made aware that his/her needs are a high priority. This helps cultivate trust between doctor and patient and facilitates future communication between both parties, as the patient is now aware that the doctors care about him/her as a person (Table 2).

**Table 2:** Second transcript about emphasising patients’ emotional needs

30	Patient	So, I will make it? Will I, really fully recover?
31	Medical Student	So far at this stage I cannot tell you that you will be 100% cured. But, we, the entire hospital, will try our best in assisting you in fighting the disease. As doctors, we cannot accurately say that we can cure every patient, but we will try our best to help you fight the disease. If you have any questions or needs, you can come to us anytime.

#### Showing insufficient care about patients’ emotional needs

Medical students show very superficial concern about patients’ emotional needs. In Table 3 (line 5), after giving the patient some distressing news, the student acknowledges the patient’s anger by showing empathy. However, the student is not assertive when comforting the patient and is cut off (line 6). The patient expresses his shock and worry for his life and family (line 6). His refusal to accept the diagnosis is a non-verbal indicator that he is not ready for further discussion and needs comforting. However, the medical student uses a rather dismissive method to show his awareness of the patient’s emotional needs (line 7). Despite expressing understanding for the patient’s situation, the medical student does not actually comfort or dissuade the patient from thinking negative thoughts as the students in Tables 1 and 2 do. The student merely expresses understanding and moves on to talking about the ‘other issues’ the patient is concerned about. This insensitive decision reflects that although the student verbally expresses concern for the patient’s emotional needs, he does not care enough to tend to them. The patient, feeling distressed that his emotional needs are not being met, lashes out at the student (line 8), asking him what he would do. This emotional outburst indicates that the patient recognises the student’s lack of caring and is not satisfied with the encounter. This situation is detrimental to future doctor–patient communication. Furthermore, it may be explained by the student showing concern out of a desire to follow protocols and frameworks (such as the SPIKES and COMFORT frameworks) that they have been taught to follow, but in fact not actually understanding the rationales behind them. Thus, the student is saying he cares as if it is something to tick off of a list instead of actually caring (Table 3).

**Table 3:** Transcript about showing insufficient care about patients’ emotional needs

5	Medical Student	Oh, I, I understand that you are angry...
6	Patient	Is this confirmed? But that is impossible, I have children and there’s so much I want to do. This can’t be happening.
7	Medical Student	Um, I understand that you have a lot to worry about and handle. So, other than this, are there any other issues that you are concerned with?
8	Patient	Obviously, the cancer. What does it mean, cancer? Does it mean I’m dying? What about my daughter? She is only two years old, who is going to take care of her? My wife is not working, what, what can I do, doctor? What would you do?

#### Prioritising the preferred treatment plan instead of respecting the needs of patients’ families

Another communication pattern observed was one in which the medical student prioritises convincing the patient’s family to accept his/her preferred treatment plan without demonstrating awareness of the preference of the patient’s family members. The dialogue in  Table 4 is one between the medical student and the patient’s child. The patient’s child asks whether he/she can visit the patient anytime they want, showing care for the patient and their strong familial bond (line 43). In addition to responding to the question directly (line 44), the student immediately presses on with his suggestion of treatment termination. The student prioritises this over other concerns, such as the emotional needs of the patient’s child. The patient’s child further expresses concern about the patient’s comfort (line 45). Although the student does elaborate somewhat on options for making the patient more comfortable, he immediately reiterates his suggestion to terminate treatment (line 46), arguing that it is not a form of giving up. The patient’s child, who is not receptive to the suggestion to terminate treatment, demonstrates his/her discomfort with the idea (line 47). This shows that the medical student is trying to encourage the patient’s child to choose treatment termination, although the child’s main concern is the comfort of his/her parent. The non-verbal cues clearly show that the child cares a lot about the patient, enough to want to visit often and to be concerned for the latter’s comfort. This is common in the Chinese context, in which filial piety is emphasised. Given the role of filial piety in Chinese society, the medical student should have understood that the patient’s child may only consider terminating treatment and losing his/her loved one when no other options are available. Although the student does answer all of the patient’s child’s questions, his repeated emphasis on a treatment option the child is uncomfortable with leaves the patient’s child unsatisfied. Perhaps the student could have put more effort into observing the patient’s child’s reaction to his suggested treatment option. The student should have avoided strongly suggesting treatment termination once the child showed discomfort and explored other treatment options. Although doctors are authority figures in Chinese society and doctor-centred approaches are the norm, doctors should not disregard the opinions of patients’ families. The decision-making process surrounding end-of-life treatment options is very family-influenced in Chinese contexts and doctors should be mindful of showing a lack of concern for families’ wishes. In this case, perhaps a less forceful method of communication would have guided the patient’s child towards the student’s suggested treatment option instead of making him/her uncomfortable (Table 4).

**Table 4:** Transcript about prioritising the preferred treatment plan

43	Patient’s Child	That means I can visit him whenever I want?
44	Medical Student	Um, we can help you arrange that. If you want to treat the patient better, there is a way to allow him to leave us with happiness, comfort and dignity, and we can help you do that.
45	Patient’s Child	Is there any way for him to feel more comfortable?
46	Medical Student	Um, we have a few ideas to help him feel more comfortable. For example, if he is in pain, we can prescribe him painkillers. If he starts coughing heavily, our nurses, occupational therapists and physical therapists can help him extract the sputum. But we would like to let you know that treatment termination doesn’t mean giving up on the patient, but helping him feel more comfortable when he leaves us.
47	Patient’s Child	Um…

#### Acknowledging dying patients’ physical discomfort and respecting their treatment preferences

In this communication pattern, some of the medical students place importance on alleviating patients’ physical discomfort and respecting their wishes. In  Table 5, the medical student asks the patient’s child how the patient felt while using the mask (line 21). This demonstrates the student’s concern for the patient’s physical comfort. Taking the patient’s child’s statement about how their father would prefer dying to wearing the uncomfortable mask (line 22) into account, the medical student’s response might also show the student’s awareness of the dying patient’s possible desire for a good death, an element of which (in Chinese culture) is ‘being free from pain and suffering’ during the end-of-life process [22]. After the patient’s child reflects on the patient’s extreme discomfort when using the oxygen mask (line 22), the doctor asks whether the child thinks his/her parent would want to continue using the machine despite the discomfort. This question shows the doctor’s respect for the patient’s preference. Given the Chinese context in which family members are usually involved in the end-of-life process and children are expected to know their parents’ wishes, the medical student here respects the judgement of the patient’s child, trusting him/her to speak on behalf of the patient. Unlike the student in  Table 4, this student does not aggressively push his/her suggested treatment method and demonstrates concern for the patient’s preferences. After the patient’s child says that the patient would not want to continue using the machine (line 24), the student immediately clarifies that although the mask causes discomfort, it is the most comfortable treatment option for the patient (line 25). The student shows his concern for the patient’s comfort and acknowledges his preference for not continuing to using the machine while simultaneously detailing treatment information for the patient so that the patient’s child can independently make an informed decision. The medical student respects the patient’s desire for a more comfortable treatment and provides his professional suggestion in order to balance fulfilling this desire and proceeding with effective treatment. This communication pattern is more effective than pattern 3 in guiding patients to accept doctors’ suggested treatment, as it demonstrates the latter’s concern and respect for the treatment preferences of patients and their family members (Table 5).

**Table 5:** Transcript about acknowledging dying patients’ physical discomfort and respecting their treatment preferences

21	Medical Student	If he removed the oxygen mask, there would be definite risks. But I would like to know more about the time when your father told you about his breathing experience when he was wearing the mask.
22	Patient’s Child	He said when he was wearing the mask, the air was plunging in and he felt very uncomfortable. He said he never wanted to wear it again, even if it means dying.
23	Medical Student	Um, (cough), if your father could communicate with us, do you think he would want to continue using the machine?
24	Patient’s Child	He, I think he wouldn’t want to. But could the machine help him?
25	Medical Student	Um, as I mentioned before, the machine is the best solution we can offer so far. Of course, there are further treatments, but I must tell you that the treatments would be more invasive than the machine and more uncomfortable for the patient. Yes, if your father felt uncomfortable with a non-invasive oxygen mask, it would be even more unbearable for him to undergo further treatment.

#### Directly disclosing bad news to patients

The medical students observed using this communication pattern directly revealed bad news to the patients without showing concern for their emotional state. Their priority was to provide information on the results rather than care for the patients’ emotional needs. In  Table 6, the medical student simply reveals the diagnosis and details of the medical examination to the patient, directly communicating the bad news without softening the delivery of such shocking news. Although the news that the patient has cancer is hard to take in because it may be fatal, the student prioritises the delivery of the information in a clear and concise manner. Understandably, the patient’s reaction involves shock, denial and worry about the impact it may have on his life (line 2). The patient’s shock may also be a reaction to the medical student’s communication method. If the student does not follow up by trying to comfort the patient, the patient’s emotional state may be severely affected, which may in turn affect the efficiency of future communication and treatment. This direct disclosure of bad news is done differently in  Table 7, where the student starts by warning the patient about the severity of the news (line 12). This helps prepare the patient to receive potentially shocking news. After the patient responds (line 13), the student asks whether the patient wants to know about his/her situation (line 14), making sure that he/she is ready to receive the bad news. After the patient confirms that they want to know the results (line 15), the student gives yet another warning (line 16), telling him/her to be mentally prepared for the bad news. Although the student does not provide any words of comfort that cater directly to the patient’s emotional needs, the student’s repeated warnings and questions show an awareness of the patient’s emotional needs. The student is aware that the bad news may not be easy for the patient to take in, but it helps lessen the impact while still delivering the news directly. Although the students in both Tables 6 and 7 deliver the bad news directly to the patient without comforting him/her or asking about the patient’s wishes, the student in Table 7 makes more effort to ensure that the patient is not shocked by the news (Tables 6 and 7).

**Table 6:** First transcript about directly disclosing bad news to patients

1	Medical Student	We extracted the pulmonary oedema in your lungs and took it for assay. We found some cancerous cells in the pulmonary oedema and diagnosed that you have lung cancer.
2	Patient	Is that confirmed? It’s not possible it could be anything else? Because it is quite impossible. I am young, right? I have children to take care of and I have a lot to do for my family. I cannot have cancer.

**Table 7:** Second transcript about directly disclosing bad news to patients

12	Medical Student	Um, actually, this piece of news might be more serious than you think.
13	Patient	Oh, how serious, doctor?
14	Medical Student	Um, I want to know if you would like to know about your situation?
15	Patient	Yes, I want to know.
16	Medical Student	Yes, but I want you to be mentally prepared for the news...

#### Addressing patients’ familial requirements

Addressing the familial needs of patients was also identified as a communication pattern involved in breaking bad news. For instance, in  Table 8, the patient expresses hope that the treatment plan will not get in the way of her daily routine, including taking care of her daughter and family (line 49). Her family role as a caregiver is clear. The medical student acknowledges this concern (line 50) and suggests daytime treatment plans to allow the patient to fulfil her family’s needs at night. In doing so, the student demonstrates understanding of the patient’s needs and shows the patient that he/she cares about her needs. Satisfied that her familial needs have been addressed, the patient agrees to the treatment arrangement (line 51). Ultimately, the medical student addresses the patient’s familial needs without delaying treatment. This puts the patient at ease and leaves her feeling emotionally satisfied, fostering the development of a better doctor–patient relationship and facilitating future communication between the two (Table 8).

**Table 8:** Transcript about addressing patients’ familial requirements

49	Patient	Um, most importantly, I hope these treatments won’t affect my daily routine. I have to take care of my daughter and family. I hope it won’t take up too much of my time.
50	Medical Student	Because, um, it is possible for some of the treatment plans to take place in the hospital (overnight). But there are a lot of plans that can be done during daytime in the hospital. That means you come in during the day and we will give you some treatment. Then you can go back home and take care of your children. So, we will try our best to cope with the needs of you and your family so you can spend more time with them.
51	Patient	Um, good, good.

## Discussion

This study examines Chinese medical students’ communication patterns when delivering bad news to patients by analysing verbal interactions during a series of scenario-based role-play learning activities. Six different communication patterns are identified. The relative success of patient-centred communication patterns (namely patterns 1, 4 and 6) echoes the literature on effective doctor–patient communication. It is important for doctors to provide emotional responses and to show concern to prevent patients from feeling alone.[23] In doing so, the medical students using patterns 1, 4 and 6 (Tables 1, 2, 5 and 8) facilitate better future doctor–patient communication. Patients’ emotional needs are addressed when doctors can spend time with them and show care for their emotional needs in addition to their medical concerns. Furthermore, the medical students using patterns 1, 4 and 6 all demonstrate awareness of the patients’ emotional or familial needs. They respect the wishes of the patients and/or their family members and use caring statements and questions to show their concern for and understanding of the patients’ needs. This helps patients feel that their needs are respected and acknowledged.

The cases above demonstrate the importance of showing empathy towards patients, tying in with the suggestion of Fan et al. that doctors should heavily emphasise showing empathy when breaking bad news despite patients prioritising facts and information [13]. Treatment plan information and details are indeed very significant for patients, as the only way to learn about their diseases is through communicating with their doctors. According to Fan et al., in Asian countries, China especially, patients perceive doctors as authoritative and professional [24]. However, patients in the Chinese context also value doctors who can address their emotional needs, family duties and expectations [24]. Communication patterns 2, 3 and 5 show the importance of addressing patients’ needs. Although patients value information and professional advice, the students using communication patterns 2, 3 and 5 mainly focus on delivering facts and neglect addressing the patients’ emotional needs. As a result, they obtain less positive patient reactions than the students prioritising the patients’ needs. The student using pattern 2 (Table 3) shows only a superficial level of care for the patients’ needs, focusing instead on delivering the information. Sensing this insincerity, the patient lashes out at the student. Ultimately, this causes the doctor–patient relationship to deteriorate and hinders future communication and treatment. The student in this case would have benefitted from expressing genuine care for the patient’s needs instead of superficially addressing them as if they were merely adhering to guidelines. The student using pattern 3 (Table 4) demonstrates disregard for the wishes of the patient and the patient’s family in favour of persuading the patient’s child to terminate treatment. Despite being a medical professional with a responsibility to deliver professional advice and information to the patient’s family, the student disregards the non-verbal cues from the patient’s child suggesting the latter’s discomfort with the idea of treatment termination. According to McHenry et al., clinician’s nonverbal communication is just as important as verbal communication during doctor-patient discourse [25]. Neglecting the cues from the patient’s child, the student continues to stress his preferred treatment plan and make the patient’s child uncomfortable. As nonverbal cues can serve as a guide for identifying patients’ emotional state, this student would have benefitted from not overwhelming the patient’s child with his professional opinion [26]. Instead, he should have spent more time observing and addressing the patient’s child’s needs and wants and demonstrating more empathy for them. Using pattern 5, both of the students in Tables 6 and 7 also focus on the delivery of bad news instead of demonstrating care for their patients’ emotional needs. Although the student in Table 7 takes more precautions against shocking the patient, both of the students prioritise delivering the information over comforting their patients. Doing so may result in emotionally distraught patient reactions, as shown by Table 6. With reference to Schapira [9], the six-step SPIKES protocol can help doctors properly prepare themselves for breaking bad news. The COMFORT model can also provide a step-by-step guide for medical staff to break bad news [8]. The medical students using the above three communication strategies should learn from these models to ensure the comfort and emotional well-being of their patients. However, it should be noted that although protocols such as SPIKES and COMFORT are useful in providing a structural guide regarding bad news delivery for medical students and staff, they should still be mindful of the cultural background of the patients instead of following the steps strictly. Moreover, bad news discussion is highly varied among individuals. Apart from doctor-patient communication, patients’ expectations on bad news conversations can be shaped by gender, age and the type of disease [12].

Better patient reactions are observed for the medical students demonstrating an understanding of the role of family members during their delivery of bad news. The students using patterns 4 and 6 (Tables 5 and 8, respectively) are aware of the relation between their patients’ and their family members. The student using pattern 4 demonstrates trust in the patient’s child’s ability to make the best decision on the patient’s behalf. Given the strong emphasis on filial piety in China, many elderly parents put great trust in their children to take care of them in old age, even giving them the power to make end-of-life decisions and create treatment plans on their behalf [24, 27, 28]. Aware of this context, the student shows respect for the child’s understanding of the patient by asking what treatment options the patient would want while still offering professional advice about treatment options. This is a good balance between the delivery of treatment information and caring for emotional needs. Furthermore, this method has better results than pattern 3, in which the student disregards the opinions of the patient’s family and pushes his preferred treatment, damaging the doctor–patient relationship as a result. The student using pattern 6 realises the patient’s familial needs and immediately suggests an alternative treatment plan to ensure that the patient can meet these needs without delaying treatment. In doing so, the student shows the patient that her needs are valued. The opposite occurs for the student using pattern 2, who delivers the bad news that the patient has cancer without considering how his familial needs and duties would affect his reception of the news. The student shows insufficient care towards these familial needs instead of truly empathising with the patient, dismissing his needs (only saying, ‘[u]m, I understand that you have much to worry and handle’) and moving on immediately to discussing treatment. As a result, the patient becomes offended, points out his familial need to provide for his daughter and asks the student what he would do. A more sensitive student would have realised that such bad news would affect the patient’s emotions regarding his family and demonstrated more concern for his needs when delivering bad news. Showing concern about patients’ familial roles and duties is perceived as a form of care and understanding. This is largely due to collectivism that is of value in the Chinese culture, as opposed to the Western ideology of individualism. Chinese patients generally prefer joint decision-making with family members; many elderly patients believe that their children understand their preferences and thus rely on their family members to decide on treatment plans or end-of-life care on their behalf [24]. Thus, it is significant for patients in the Chinese contexts to be able to continue their duties as parents or children even while they are undergoing treatment, and clinicians treating Chinese patients should be aware of this and make necessary adjustments in the process of treatment. As a result, doctors who demonstrate more awareness and respect for the role of family in end-of-life decisions tend to be better received in the Chinese context.

## Conclusions

This study examines final-year medical students’ communication pattern for delivering bad news to patients in the Chinese context. The six communication patterns identified include how medical students communicate with patients, such as how they deliver bad news, direct patients to their primary concerns, explain treatment plans and build rapport with patients. They also include the kinds of question types and turn-taking patterns that medical students use when communicating with patients. The role-playing exercise allowed for an in-depth investigation of medical students’ communication styles when breaking bad news. A majority of the students used the much-preferred patient-oriented approach to cater to the patients’ emotional and factual needs. This approach can enhance trust and improve the relationship between doctors and patients. The role of family is also emphasised in this approach, with the medical students often informing and acknowledging the patients’ family members. Acknowledgement about Chinese cultural norms are also demonstrated by successful exchanges, indicating the importance of cultural awareness while breaking bad news. However, more training on delivering bad news is needed to teach medical students how to be more sensitive to non-verbal cues, such as tone, manner and attitude. Breaking bad news and having end-of-life conversations are inevitable for medical students. The above-mentioned approaches and topics should be included in the future medical education curriculum and training in order to teach medical students and staff to enhance their cultural awareness during communication with patients. As such, additional training would allow future physicians to experience and better prepare for these elements of clinical work.

## Data Availability

The datasets generated and/or analysed during the current study are not publicly available due to the confidentiality agreements approved by the Joint Chinese University of Hong Kong – New Territories East Cluster Clinical Research Ethics Committee.
